# *Ridleyandra iminii* (Gesneriaceae), a new species from Peninsular Malaysia

**DOI:** 10.3897/phytokeys.19.4064

**Published:** 2012-12-28

**Authors:** Mat Yunoh Siti-Munirah

**Affiliations:** 1 Forest Research Institute Malaysia, 52109 Kepong, Selangor, Malaysia

**Keywords:** Gesneriaceae, *Ridleyandra*, Peninsular Malaysia, new species

## Abstract

A new endemic species of *Ridleyandra* (Gesneriaceae), *Ridleyandra iminii* Siti-Munirah from Peninsular Malaysia is described and illustrated. Among *Ridleyandra* species, it is the only one with a dark red flower.

## Introduction

During a botanical collecting trip in 2008 to Gunung Benom, Pahang, Peninsular Malaysia, a new species of *Ridleyandra* was discovered: here described as *Ridleyandra iminii* Siti-Munirah. In Peninsular Malaysia, currently 12 species of *Ridleyandra* are known (Weber 1998; Kiew 2009). This is the first *Ridleyandra* species with deep red flowers to be described. Although in vegetative characters it resembles *Ridleyandra morganii* (Franch.) A.Weber, it is completely different in the colour and patterning of the flower. The inside of the purple corolla tube of *Ridleyandra morganii* has white lines on the lower surface of the throat, and the lobes are concolorous with the outside of the tube. In contrast, the corolla lobes and throat of *Ridleyandra iminii* are red and contrast with the white outer surface of the tube, and the throat lacks contrasting lines. Weber has drawn attention on the coloration of *Ridleyandra* species. Flowers of other *Ridleyandra* species are yellow, white, blue or violet so the deep red throat is remarkable.

## Taxonomy

### Ridleyandra iminii

Siti-Munirah sp. nov.

urn:lsid:ipni.org:names:77123887-1

http://species-id.net/wiki/Ridleyandra_iminii

Figure 1

#### Diagnosis.

*Ridleyandra iminii* is most similar to *Ridleyandra morganii*  (Franch.) A.Weber in its dentate leaves with blunt teeth more than 5 mm long, but it differs in its shorter peduncles (not 7–10 cm long), the deep red colouration of the corolla (not deep purple with white lines in the throat) and longer capsules (5-6 cm long, not 4.5–5 cm long).

#### Type.

Peninsular Malaysia.** **Pahang, Gunung Benom, Krau Game Reserve. 8 January 2008 (fl & fr), Siti-Munirah FRI 55387 (holotype: KEP!).

#### Description.

Perennial herb, **stem **unbranched, woody, to 25 cm long, glabrous except for dense dark uniseriate, multicellular hairs, ca 0.5 mm long, on upper portion of petioles, on the lower surface of midrib and on peduncles. **Leaves **opposite, clustered in a rosette at the top of the stem; petioles 1–4 cm long; lamina lanceolate-oblong, 9–18.5 × 3–5.5 cm, glossy above, slightly paler beneath, base attenuate, margin undulate, very coarsely serrate, teeth to nearly 1 cm long, broad and blunt, apex acute; midrib and veins impressed above, prominent beneath, lateral veins (10–)12(–16) pairs. **Inflorescence** single-flowered, peduncle slender, pale green, 5–8 cm long; bract pair lanceolate, 2–3 × 1–2 mm; pedicels 2.5–3 cm; *sepals* light green, divided to base, lanceolate, 5–7 × ca. 1 mm, apex acute; *corolla *trumpet-shaped; tube white outside, dark red within, ca. 4 cm long, ca. 5 mm wide at base dilating to 10–15 mm wide at the mouth, outside finely pubescent, throat and lobes dark red, nectar guides raised and slightly darker, inner surface of throat finely velvety; lobes 5, upper two lobes reflexed, ca. 5 x 10 mm and lower three lobes extending beyond the upper, ca. 5 × 12 mm; *stamens* with filaments 2–2.5 cm long, anthers white, ca. 1 × 1 mm, connective small and horn-like, staminode vestigial; *ovary* and style ca. 3 cm long, stigma broadly triangular, white. **Capsules** curved downwards, glabrous, 5–6 cm long, ca. 3 mm thick, sepals not persisting.

**Figure 1.**
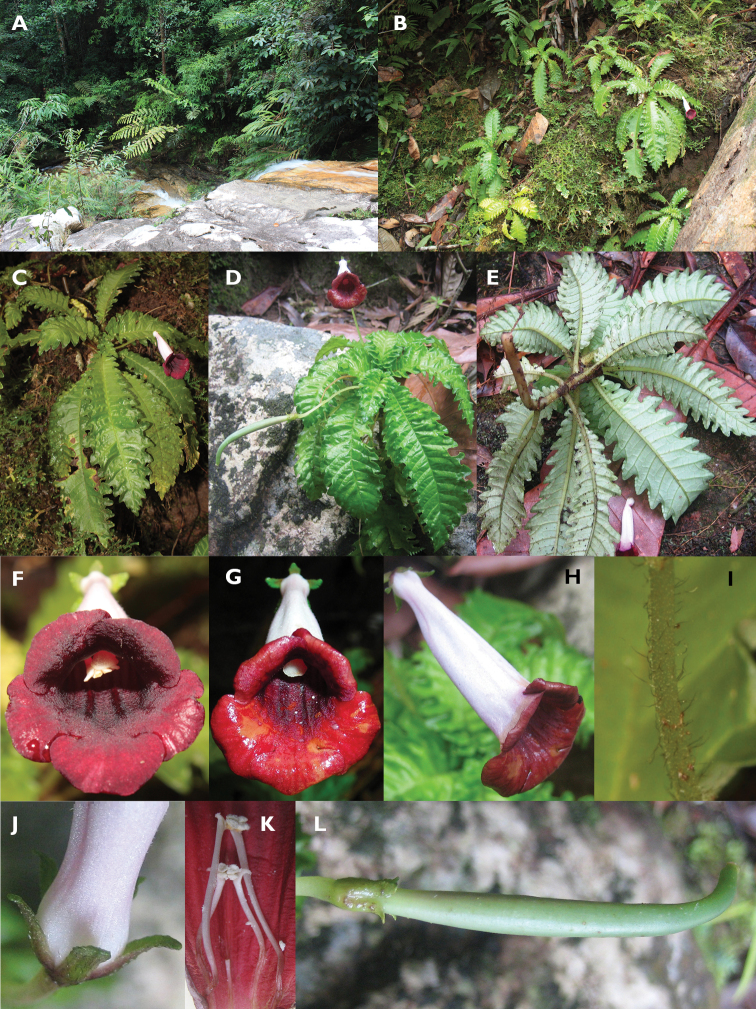
*Ridleyandra iminii*. **A, B** habitat **C–E** habit **F** flower with mature stamens **G** flower with mature stigma **H** side view of flower **I** peduncle hairs **J** sepals **K** stamens and staminode **L** fruit. (Photos: **A–C, F** by K. Imin, **D–E, G–L** by M.Y. Siti-Munirah).

#### Distribution:

*Ridleyandra iminii* is known only from the type locality, Peninsular Malaysia. Pahang: Gunung Benom, Krau Game Reserve, 3°45'N, 102°19'E.

#### Ecology.

In upper hill dipterocarp forest on wet, sandy, moist soil on a shaded, steep slope and river bank at ca. 700 m altitude.

#### Etymology.

The species is named after Mr. Imin Kamin, research assistant and plant collector in the Kepong Herbarium (KEP), Forest Research Institute Malaysia, with whom I first collected the plant. **Conservation status**. Rare (RA). The Malaysian Rare category has the following definition: the taxon is not exposed to any known direct or plausible potential threat and does not qualify under the five IUCN criteria and it occurs in ≤ 2 sites or has an EOO (extent of occurrence) ≤ 100 km2 or AOO (area of occupancy) ≤ 10 km2 (Chua 2012). In the case of this species, although it occurs in a Totally Protected Area (an area that is legally protected), it still vulnerable because it lies beside the main tourist trail and its population numbers about 200 individuals.

#### Specimens examined.

 Peninsular Malaysia. Pahang: Benom, Krau Game Reserve, 15 November 2009 (fl), A.R. Ummul-Nazrah FRI 70717 (KEP).

## Supplementary Material

XML Treatment for Ridleyandra iminii

## References

[B1] Chua LSL (2012) Conservation. In: Kiew R, Chung RCK, Saw LG, Soepadmo E (Eds) Flora of Peninsular Malaysia 2, 3: 3–10

[B2] Weber A (1998) (‘1997’) Revision of the genus *Ridleyandra* (Gesneriaceae). Beiträge zur Biologie der Pflanzen 70: 225−273.

[B3] Kiew R (2009) Three New Species of Gesneriaceae from Kelantan. Gard. Bull. Singapore 61(1): 73−79.

